# Leber Hereditary Optic Neuropathy With Magnetic Resonance Imaging Findings Suggestive of Optic Perineuritis and Optic Neuritis: A Diagnostic Challenge

**DOI:** 10.7759/cureus.80439

**Published:** 2025-03-11

**Authors:** Kazuhiro Horiuchi, Shuntaro Nakamura, Kazuki Yamada

**Affiliations:** 1 Neurology, Hakodate Municipal Hospital, Hakodate, JPN

**Keywords:** genetic testing, leber hereditary optic neuropathy, mitochondrial disease, optic neuritis, optic perineuritis

## Abstract

Leber hereditary optic neuropathy (LHON) is a rare mitochondrial disorder characterized by subacute, painless, and bilateral vision loss, typically affecting young men. LHON is caused by mitochondrial DNA mutations, most commonly m.11778G>A, m.14484T>C, and m.3460G>A. LHON has incomplete penetrance, with a higher prevalence in men, and its diagnosis is often delayed because of clinical overlap with other optic nerve disorders, such as optic neuritis.

Herein, we report the case of a 37-year-old man presenting with progressive vision loss in both eyes over two months. Early magnetic resonance imaging (MRI) findings were suggestive of optic neuritis or peripapillary optic neuritis. Based on the MRI findings, the differential diagnoses for the patient's condition included multiple sclerosis, neuromyelitis optica spectrum disorders, anti-myelin oligodendrocyte glycoprotein (MOG) antibody-related diseases, sarcoidosis, Behçet's disease, systemic lupus erythematosus, Sjögren's syndrome, and idiopathic optic neuritis and peripapillary optic neuritis. The patient was treated with intravenous methylprednisolone and plasmapheresis. Despite immunotherapy, the patient's symptoms worsened. Comprehensive evaluation revealed normal cerebrospinal fluid and negative autoimmune markers. Mitochondrial DNA testing confirmed the m.11778G>A mutation, which led to the diagnosis of LHON. The patient was treated with ubidecarenone because of the unavailability of idebenone; however, no significant visual improvement occurred. His vision stabilized at 0.3 in the right eye, whereas the left eye became completely blind.

This case highlights the diagnostic challenges of LHON, particularly when MRI findings mimic optic neuritis. The preservation of the pupillary light reflex and nonresponse to immunotherapy are key diagnostic clues. Early genetic testing is crucial in cases with atypical progression to confirm LHON and guide management. This case underscores the need for heightened awareness of the incidence of LHON in patients with subacute vision loss unresponsive to conventional treatments.

## Introduction

Leber hereditary optic neuropathy (LHON) is a rare, maternally inherited mitochondrial disorder that primarily affects young adults, typically men in the second and third decades of life. LHON is characterized by subacute, painless, and bilateral vision loss and is most often caused by mitochondrial DNA (mtDNA) mutations, specifically in the genes encoding subunits of complex I of the respiratory chain; the most common mutations are m.11778G>A, m.14484T>C, and m.3460G>A [1,2]. A recent nationwide questionnaire survey conducted in Japan estimates the prevalence of LHON to be one in 50,000. LHON has traditionally been considered a disease affecting young men, with a peak incidence between the ages of 14 and 26 years and a male-to-female ratio of 5:1. However, recent studies suggest that the ratio is closer to 3:1 and approaches 1:1 after the third decade of life. Furthermore, although the disease is more common in young adults, it can present at any age, with 10% of cases having onset after the age of 50 [3,4]. Although the disease typically results in irreversible vision loss before the age of 50, its diagnosis can often be delayed because of its clinical resemblance to other optic nerve disorders, particularly optic neuritis (ON), which complicates early detection and intervention [1,2].

Among LHON families, 85%-90% of carriers are homoplasmic for the mtDNA mutation (i.e., 100% mutant), while the remaining individuals are heteroplasmic, carrying a mixture of both wild-type and mutant mtDNA species. The risk of disease conversion is significantly lower if the mutational load remains below the critical threshold of 60% [5].

Despite extensive research, the pathophysiology of LHON remains incompletely understood. It is widely accepted that mitochondrial dysfunction, particularly involving complex I, is crucial to disease onset; however, the exact mechanisms by which these mutations cause optic nerve degeneration remain unclear [6]. LHON is a mitochondrial disorder primarily caused by mutations in mtDNA, which predominantly affect retinal ganglion cells (RGCs). These cells, located in the inner retina, are highly energy-dependent due to their elevated mitochondrial content and reliance on oxidative phosphorylation (OXPHOS) for adenosine triphosphate (ATP) production. Mutations in NADH dehydrogenase (complex I) disrupt the electron transport chain, leading to impaired ATP synthesis and excessive production of reactive oxygen species. This dysfunction results in RGC degeneration and subsequent visual loss. Among these mutations, the ND4 mutation is the most prevalent and is associated with the most severe prognosis. Research efforts have focused on restoring ATP production in complex I-deficient mitochondria, and animal models of complex I inhibition have been instrumental in testing potential therapeutic interventions aimed at mitigating RGC loss in LHON [1]. Furthermore, recent research has indicated a potential relationship between LHON and other systemic disorders, including neurological conditions such as multiple sclerosis. This has prompted inquiries into the relationship between mitochondrial disturbances and demyelinating diseases [7]. However, the full spectrum of clinical manifestations and the role of extraocular involvement in patients with LHON remain topics of debate.

Herein, we report the case of a 37-year-old man who developed progressive vision loss over two months, with magnetic resonance imaging (MRI) findings suggestive of optic perineuritis and ON. Despite receiving immune therapy, the patient did not respond to treatment, which led to further genetic testing. The genetic analysis ultimately confirmed LHON. This report aimed to highlight the diagnostic challenges associated with LHON, particularly when it presents with MRI findings that overlap with other optic nerve disorders. This case emphasizes the importance of timely genetic testing in similar cases to avoid misdiagnosis and initiate appropriate management.

## Case presentation

The patient was a 37-year-old man diagnosed with LHON. The patient had a history of intellectual disability and autism spectrum disorder, for which he was taking risperidone 0.5 mg daily. He managed his activities of daily living on his own and lived independently. He had no family history of neurological diseases, vision loss suggestive of LHON in his parents or other relatives, allergies to foods or medications, or excessive alcohol consumption or smoking. He also had no history of prior surgeries.

Two months before hospitalization, the patient reported prolonged study sessions during which he had strained his eyes. Shortly after, he noticed a gradual decline in vision in his left eye. He sought consultations at an ophthalmology and neurology clinic at another hospital; however, no abnormalities were found, and he was advised to follow up. One week before admission, he noticed a decrease in vision in his right eye, prompting another visit to an ophthalmology clinic. Swelling of the optic disc was noted, and he was referred to our neurology department with a suspicion of ON.

Upon examination, there were no abnormalities detected during the general examination and the patient was vitally stable. No eye, eyelid, or conjunctival abnormalities were observed. His lung sounds were clear, and the abdomen was nontender with no skin rashes or edema. No palpable lymphadenopathy was noted. On neurological examinations, the left eye exhibited a blind spot in the upper visual field, with the lower visual field showing only hand motion perception. The visual acuity in the right eye was also decreased at 0.3 (6/18). The patient experienced mild pain in both eyes. Both eyes showed normal direct and consensual pupillary light reflexes. The pupil size was 3 mm in both eyes, and no difference was noted between the eyes. No abnormalities were observed in other cranial nerve examinations or motor, sensory, and cerebellar systems.

**Figure 1:**
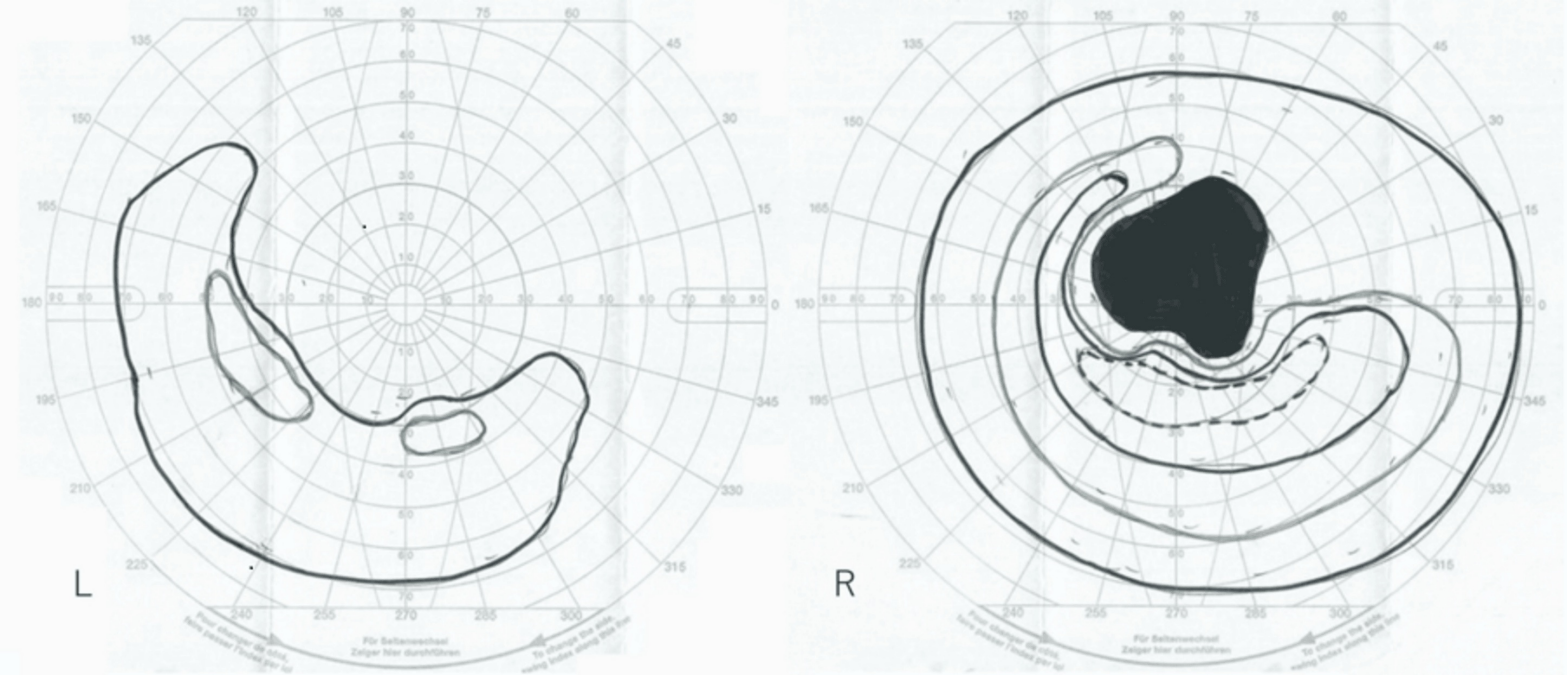
Goldmann visual field testing Goldmann visual field testing revealed central scotomas and nasal and inferior visual field defects in the right eye, along with extensive defects in the central, nasal, and superior visual fields in the left eye. L: left eye; R: right eye

Goldmann visual field testing revealed central scotomas and nasal and inferior visual field defects in the right eye, as well as extensive defects in the central, nasal, and superior visual fields in the left eye (Figure 1). Blood tests, including complete blood count and routine biochemistry, were unremarkable, and no inflammatory markers or glucose metabolism abnormalities were found. Autoimmune markers (SS-A, myeloperoxidase-specific antineutrophil cytoplasmic antibody, and proteinase 3-specific antineutrophil cytoplasmic antibody) were negative. Further testing revealed negative anti-aquaporin-4 and anti-myelin oligodendrocyte glycoprotein (MOG) antibodies. Cerebrospinal fluid (CSF) analysis showed an opening pressure of 60 mmH_2_O and a closing pressure of 40 mmH_2_O, with a cell count of 0/µL, protein level of 56.1 mg/dL, and immunoglobulin G index of 0.56. Oligoclonal bands were absent, CSF cytology was class I, and cultures were negative.

**Figure 2:**
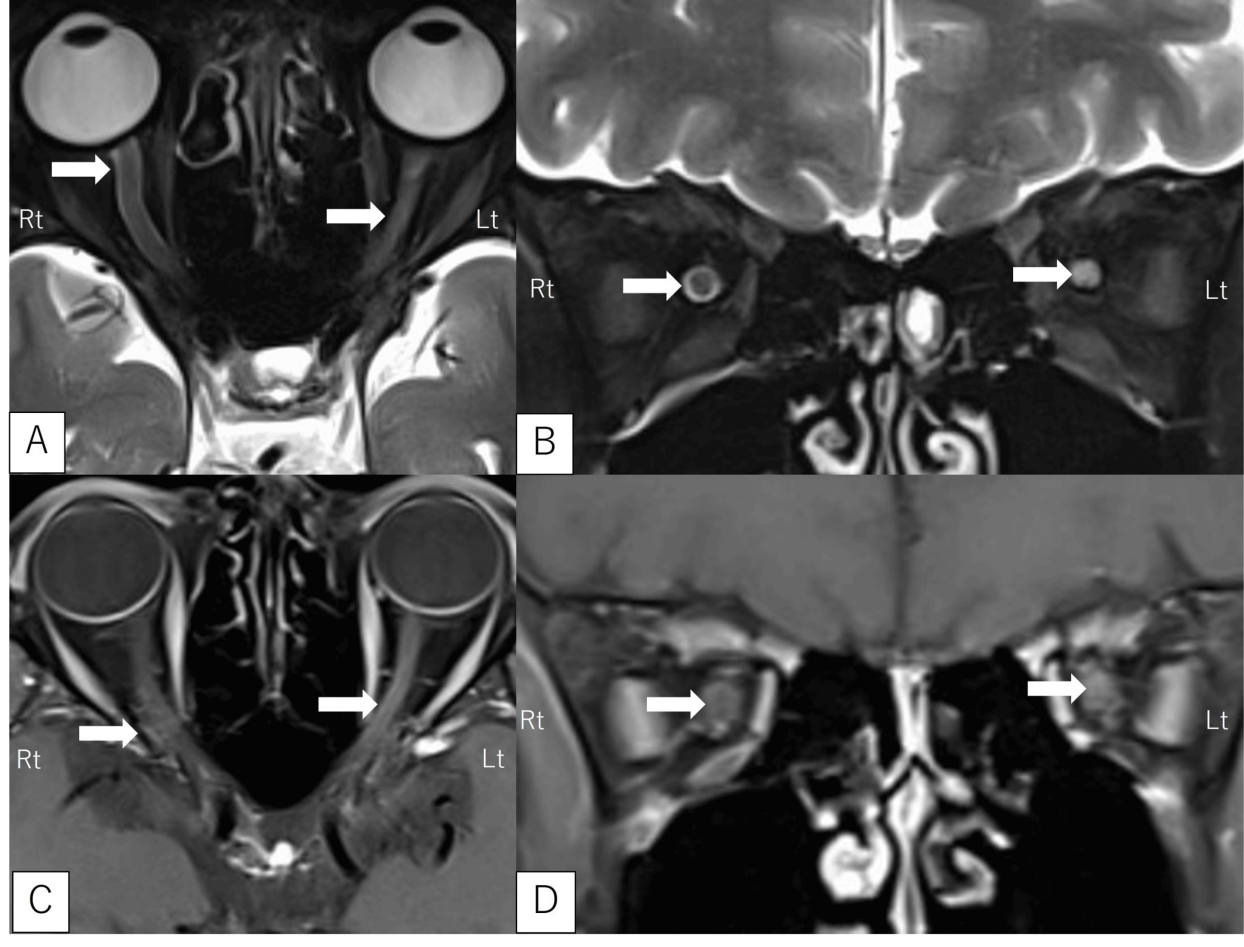
Orbital magnetic resonance imaging (MRI) findings: (A) axial and (B) coronal: short-tau inversion recovery (STIR); (C) axial and (D) coronal: gadolinium-enhanced T1WI On the right optic nerve, STIR images demonstrated high signal intensity in the perineural region of the optic nerve (A,B, arrow), and T1-weighted images with gadolinium contrast enhancement showed mild enhancement in the same area (C,D, arrow). These findings are suggestive of optic perineuritis. On the left optic nerve, STIR images revealed high signal intensity within the optic nerve itself, and T1-weighted images with gadolinium contrast demonstrated mild enhancement in the corresponding area (arrow), suspected of optic neuritis. MRI showed a suspicious finding of optic perineuritis in the right eye and optic neuritis in the left eye; however, they did not respond to immunotherapy, and a diagnosis of Leber hereditary optic neuropathy was made based on genetic testing. T1WI: T1-weighted imaging

The brain MRI was normal, with no abnormalities in the white matter. However, orbital MRI using short-tau inversion recovery (STIR) sequences showed a high signal intensity in the left optic nerve and a mild high signal around the right optic nerve. Gadolinium-enhanced T1-weighted imaging revealed enhancement in the left optic nerve corresponding to the high signal area, and mild enhancement was observed around the right optic nerve (Figure 2). Whole-body computed tomography revealed no malignant tumors, and lymphadenopathy suggestive of sarcoidosis or lymphoma was not noted. Visual evoked potentials, optical coherence tomography, and fluorescein fundus angiography were not performed.

Based on the findings, the patient was diagnosed with left ON and right optic perineuritis. Given that both eyes were affected, metabolic optic neuropathies were considered in the differential diagnosis. However, there was no evidence to suggest any potential contributing factors, such as nutritional deficiencies (including deficiencies of vitamins B1 and B12, folate, vitamin B6, or vitamin E), exposure to toxic substances (such as lead, methanol, ethylene glycol, chloramphenicol, digoxin, ethambutol, or isoniazid), or other metabolic disorders (including diabetes, renal failure, thyroid disease, or liver disease). Early MRI findings were suggestive of ON or optic perineuritis. Based on the MRI findings, the differential diagnoses for the patient's condition included multiple sclerosis, neuromyelitis optica spectrum disorders, anti-MOG antibody-related diseases, sarcoidosis, Behçet's disease, systemic lupus erythematosus, Sjögren's syndrome, and idiopathic ON and optic perineuritis. Treatment with methylprednisolone pulse therapy (1 g daily for three days) was initiated twice, followed by five sessions of plasmapheresis. Oral prednisolone was tapered from 40 mg/day. Despite these treatments, the patient’s symptoms did not improve, and vision continued to deteriorate. Given the lack of response to immunotherapy and the absence of visual improvement, LHON was considered as a potential diagnosis. To further investigate, CSF testing was conducted to assess for elevated lactate and pyruvate levels, which are characteristic of mitochondrial disorders. The lactate level in the CSF (stored prior to treatment) was 25.7 mg/dL (normal CSF lactate is <16 mg/dL), pyruvate was 1.63 mg/dL, and the lactate/pyruvate ratio was 15.8, suggesting mitochondrial disease. mtDNA testing (Invader assay (Life Technologies, Carlsbad, CA, US)) using blood samples revealed a mtDNA mutation (11778G>A), confirming the diagnosis of LHON. mtDNA genetic testing was performed at BML Inc. in Tokyo, and only a report of an abnormal point mutation was received (no results for mutated subunits have been reported). Heteroplasmy levels were not measured. It is well established that mutations in the MT-ND4 subunit are commonly associated with LHON, and the level of heteroplasmy is an important factor in assessing the severity of the disease. However, unfortunately, the results provided by the testing company only indicated the presence of a mutation based on the Invader assay, without specifying which subunit was affected or providing information on the heteroplasmy level.

Oral prednisolone was discontinued, and the patient was started on ubidecarenone 30 mg/day (as idebenone was unavailable in Japan). However, no vision improvement was observed. Ultimately, the left eye became completely blind, and the right eye vision remained at 0.3. The patient was discharged with stable vision, and the mild eye pain had resolved. Six months after discharge, the patient’s vision remained unchanged despite continued ubidecarenone.

We recommended that the patient receive low-vision care to help manage his visual impairment. Although he was living alone, we facilitated environmental modifications to ensure that he could receive adequate support from his family. During his hospitalization, he underwent comprehensive rehabilitation, which included environmental adjustments to enhance his daily functioning and accommodate his visual limitations.

Furthermore, genetic counseling was provided to both the patient and his family to discuss the hereditary aspects of his condition. While there were no other affected family members, genetic testing was offered. However, as the family did not wish to proceed with genetic testing, no such testing was performed for them.

## Discussion

This report presents a patient with subacute vision loss in the left eye, followed by progressive vision loss in the right eye. MRI raised suspicion of ON or optic perineuritis, leading to immunotherapy; however, the response was poor, and the patient was ultimately diagnosed with LHON. It has been reported that LHON patients with the 11778G>A mutation found in this patient have a worse prognosis for vision compared to other mutations [8].

The onset of LHON is influenced by environmental factors in addition to mtDNA mutations, with alcohol consumption and smoking being well-established risk factors [9]. Nutritional deficiencies [10], use of antibiotics (such as macrolides, aminoglycosides, and ethambutol), and antiretroviral medications [11] have also been reported as potential triggers for disease onset. In this case, the patient’s prolonged visual strain from the extensive study could have contributed to the onset of LHON, and the underlying genetic abnormality possibly served as a predisposing factor.

LHON is characterized by the preservation of the pupil light reflex and the absence of abnormalities in pupillary light responses, a unique feature that differentiates it from ON. In the present case, both pupillary light reflexes remained intact, which was atypical for ON. This finding is consistent with LHON because it suggests the preservation of melanopsin-expressing RGCs, which are involved in the pupil light reflex. Even in cases presenting with ON-like progression and findings, the preservation of the pupil light reflex and RAPD should prompt the consideration of LHON as a differential diagnosis [12].

Regarding CSF analysis, in acute classical LHON, brain MRI typically shows no abnormalities, with no evidence of demyelinating lesions in the white matter, which helps differentiate it from ON associated with demyelinating diseases. Although MRI often shows no abnormal signals in the optic nerve, there have been reports of such findings in LHON cases [13-15], highlighting the importance of considering LHON in the differential diagnosis when MRI shows optic nerve or peripapillary abnormalities. Immunotherapy may initially be administered based on the suspicion of immune-mediated ON or optic perineuritis; however, if there is no response, genetic testing should be considered to confirm the diagnosis.

Treatment for LHON includes coenzyme Q10 formulations, which demonstrate antioxidant properties and can improve mitochondrial function. However, their absorption is limited by their high lipophilicity. Idebenone, a short-chain analog of coenzyme Q10, has been developed to enhance mitochondrial delivery and can cross the blood-brain barrier. In a multicenter, randomized, placebo-controlled trial involving 85 patients, idebenone at a daily dose of 900 mg for 24 weeks did not show a significant difference in final visual acuity [16]. Another study supported the stability and effectiveness of idebenone treatment in LHON [17]. Unfortunately, in this case, idebenone was unavailable in Japan, and ubidecarenone was used instead; however, no significant improvement was observed.

Gene therapy has also been reported as a promising treatment option. Gene therapy involves the use of adeno-associated virus (AAV) vectors to deliver normal mtDNA into retinal cells, and clinical trials have shown improvement in visual function in patients with the m.11778G>A mutation. A recent study demonstrated that unilateral gene therapy could result in bilateral visual improvement, and long-term follow-up has shown sustained improvements in best-corrected visual acuity [18]. Considering the patient’s m.11778G>A mutation, gene therapy using AAV vectors may be a viable treatment option in this case.

## Conclusions

This case highlights the diagnostic challenges of LHON, especially when MRI findings mimic ON or optic perineuritis. Initially, MRI showed a high signal in the optic nerve, leading to suspicion of an immune-mediated process. However, the lack of response to immunotherapy prompted further evaluation, ultimately leading to a diagnosis of LHON through genetic testing. Notably, the preservation of the light reflex despite visual loss is a key clinical clue. When immunotherapy fails to improve suspected ON or optic perineuritis, clinicians should consider LHON and pursue genetic testing.

## References

[1] Hage R, Vignal-Clermont C (2021). Leber hereditary optic neuropathy: review of treatment and management. Front Neurol.

[2] Nikoskelainen EK, Huoponen K, Juvonen V, Lamminen T, Nummelin K, Savontaus ML (1996). Ophthalmologic findings in Leber hereditary optic neuropathy, with special reference to mtDNA mutations. Ophthalmology.

[3] Takano F, Ueda K, Godefrooij DA (2022). Incidence of Leber hereditary optic neuropathy in 2019 in Japan: a second nationwide questionnaire survey. Orphanet J Rare Dis.

[4] Poincenot L, Pearson AL, Karanjia R (2020). Demographics of a large international population of patients affected by Leber's hereditary optic neuropathy. Ophthalmology.

[5] Yu-Wai-Man P, Chinnery PF (2011). Leber hereditary optic neuropathy-therapeutic challenges and early promise. Taiwan J Ophthalmol.

[6] Harding AE, Sweeney MG, Miller DH (1992). Occurrence of a multiple sclerosis-like illness in women who have a Leber's hereditary optic neuropathy mitochondrial DNA mutation. Brain.

[7] Alorainy J, Alorfi Y, Karanjia R, Badeeb N (2024). A comprehensive review of Leber hereditary optic neuropathy and its association with multiple sclerosis-like phenotypes known as Harding's disease. Eye Brain.

[8] Oostra RJ, Bolhuis PA, Wijburg FA, Zorn-Ende G, Bleeker-Wagemakers EM (1994). Leber's hereditary optic neuropathy: correlations between mitochondrial genotype and visual outcome. J Med Genet.

[9] Kirkman MA, Yu-Wai-Man P, Korsten A (2009). Gene-environment interactions in Leber hereditary optic neuropathy. Brain.

[10] Spruijt L, Kolbach DN, de Coo RF, Plomp AS, Bauer NJ, Smeets HJ, de Die-Smulders CE (2006). Influence of mutation type on clinical expression of Leber hereditary optic neuropathy. Am J Ophthalmol.

[11] Pilz YL, Bass SJ, Sherman J (2017). A review of mitochondrial optic neuropathies: from inherited to acquired forms. J Optom.

[12] Moura AL, Nagy BV, La Morgia C (2013). The pupil light reflex in Leber's hereditary optic neuropathy: evidence for preservation of melanopsin-expressing retinal ganglion cells. Invest Ophthalmol Vis Sci.

[13] Newman NJ, Lott MT, Wallace DC (1991). The clinical characteristics of pedigrees of Leber's hereditary optic neuropathy with the 11778 mutation. Am J Ophthalmol.

[14] Blanc C, Heran F, Habas C, Bejot Y, Sahel J, Vignal-Clermont C (2018). MRI of the optic nerves and chiasm in patients with Leber hereditary optic neuropathy. J Neuroophthalmol.

[15] Arianti A, Chuman H, Kajihara N, Sakamoto N, Nao-IM.D N (2018). Atypical clinical and neuroimaging findings in Leber’s hereditary optic neuropathy: a case report. JOJ Ophthal.

[16] Klopstock T, Yu-Wai-Man P, Dimitriadis K (2011). A randomized placebo-controlled trial of idebenone in Leber's hereditary optic neuropathy. Brain.

[17] Klopstock T, Metz G, Yu-Wai-Man P (2013). Persistence of the treatment effect of idebenone in Leber's hereditary optic neuropathy. Brain.

[18] Biousse V, Newman NJ, Yu-Wai-Man P (2021). Long-term follow-up after unilateral intravitreal gene therapy for Leber hereditary optic neuropathy: the RESTORE study. J Neuroophthalmol.

